# Exploring factors influencing conversion from mild cognitive impairment to Alzheimer's Disease with time‐to‐event data and longitudinal measures

**DOI:** 10.1002/alz70860_097691

**Published:** 2025-12-23

**Authors:** Tahera Ahmed, Ping Zhang, Kuldeep Kumar

**Affiliations:** ^1^ Kids Research Institute, Perth, Western Australia, Australia; ^2^ Griffith University, Gold Coast, QLD, Australia; ^3^ Bond University, Gold Coast, QLD, Australia

## Abstract

**Background:**

Alzheimer's disease (AD) is a prevalent neurodegenerative condition primarily impacting the elderly population. Despite its widespread occurrence, the precise etiological factors remain elusive, emphasizing the critical need for early detection and intervention to mitigate disease progression. This study investigates the role of various lifestyle and behavioral factors in the progression from Mild Cognitive Impairment (MCI) to Alzheimer's Disease (AD) using advanced survival analysis techniques.

**Method:**

Data from the Alzheimer's Disease Neuroimaging Initiative (ADNI) were utilized to investigate the progression from Mild Cognitive Impairment (MCI) to Alzheimer's Disease (AD). The dataset included a total of 848 MCI individuals, of whom 336 converted to AD at different time points, while 512 did not. Time‐to‐event data and longitudinal measures were analyzed using Cox proportional hazard models, time‐dependent covariates Cox models, and Aalen's additive regression models. The analysis examined socio‐demographic, neuropsychological, behavioral, and lifestyle‐related factors, with a particular focus on novel predictors such as sleep disorders and eating disorders.

**Result:**

The analysis revealed that daily functional activity scores and sleep disorders were significant predictors of progression from MCI to AD. These factors, along with other socio‐demographic and lifestyle variables, were found to substantially increase the risk of AD over time. Comparative analyses of the survival models highlighted the unique contributions of Cox regression and Aalen's additive hazards models in identifying time‐dependent and non‐linear effects of these variables.

**Conclusion:**

This study emphasizes the importance of behavioral and lifestyle factors, particularly sleep and daily functional activities, in predicting the transition from MCI to AD.

